# Recombination, chromosome number and eusociality in the Hymenoptera

**DOI:** 10.1111/jeb.12543

**Published:** 2015-01-06

**Authors:** L Ross, H Blackmon, P Lorite, V E Gokhman, N B Hardy

**Affiliations:** * School of Biological Sciences, Institute of Evolutionary Biology, University of EdinburghEdinburgh, UK; † Department of Biology, University of TexasArlington, TX, USA; ‡ Department of Experimental Biology, University of JaénJaén, Spain; § Botanical Garden, Moscow State UniversityMoscow, Russia; ¶ Department of Entomology and Plant Pathology, Auburn UniversityAuburn, AL, USA

**Keywords:** colony size, eusociality, Hymenoptera, karyotype, recombination, relatedness

## Abstract

Extraordinarily high rates of recombination have been observed in some eusocial species. The most popular explanation is that increased recombination increases genetic variation among workers, which in turn increases colony performance, for example by increasing parasite resistance. However, support for the generality of higher recombination rates among eusocial organisms remains weak, due to low sample size and a lack of phylogenetic independence of observations. Recombination rate, although difficult to measure directly, is correlated with chromosome number. As predicted, several authors have noted that chromosome numbers are higher among the eusocial species of Hymenoptera (ants, bees and wasps). Here, we present a formal comparative analysis of karyotype data from 1567 species of Hymenoptera. Contrary to earlier studies, we find no evidence for an absolute difference between chromosome number in eusocial and solitary species of Hymenoptera. However, we find support for an increased rate of chromosome number change in eusocial taxa. We show that among eusocial taxa colony size is able to explain some of the variation in chromosome number: intermediate-sized colonies have more chromosomes than those that are either very small or very large. However, we were unable to detect effects of a number of other colony characteristics predicted to affect recombination rate – including colony relatedness and caste number. Taken together, our results support the view that a eusocial lifestyle has led to variable selection pressure for increased recombination rates, but that identifying the factors contributing to this variable selection will require further theoretical and empirical effort.

## Introduction

Sexual reproduction is near ubiquitous among multicellular life (Maynard-Smith, 1978; Bell, 1983). Recombination – the reshuffling of genomes during meiosis – is thought to be the main benefit of sex, as it increases the efficiency of selection (Otto, 2009). However, in the short term, it can also reduce organismal fitness by breaking up coadapted gene complexes (Agrawal, 2006; Otto, 2009). Theories for the evolution of recombination therefore aim to reconcile these two opposing evolutionary forces (Otto & Lenormand, 2002). In each generation, genetic variation is produced by two separate mechanisms: independent assortment (via random segregation of homologous chromosomes) during meiosis and crossing over between homologous chromosomes. Interestingly, observed recombination rates can vary dramatically, even between closely related species (White, 1973; Wilfert *et al*., 2007; Smukowski & Noor, 2011). Theory has often been successful in predicting under which circumstances selection would increase recombination rates, but statistical tests of these theories remain scarce (see however Lenormand & Dutheil, 2005).

One factor that might promote high rates of recombination is eusociality. In eusocial societies, workers forego their own reproduction to help raise their siblings. The resulting colonies can range in size from a few individuals to millions, most of them sterile workers that are highly related, usually full or half-siblings. High relatedness is thought to be crucial for the evolution of eusociality (Boomsma, 2009), but high relatedness can also be problematic for those societies. Living in a dense aggregate of close kin makes eusocial populations vulnerable to parasites (Kraus & Page, 1998; Schmid-Hempel, 1998; Schmid-Hempel & Crozier, 1999; Wilson-Rich *et al*., 2009). Indeed, there is strong empirical evidence from ants, honeybees and bumblebees that colonies with higher genetic diversity are better able to resist parasites (Shykoff & Schmid-Hempel, 1991; Baer & Schmid-Hempel, 1999; Tarpy, 2003; Hughes & Boomsma, 2004). Genetic diversity might also be important for division of labour (Oldroyd & Fewell, 2007; Wilfert *et al*., 2007). Many eusocial colonies display extreme phenotypic and behavioural diversity among and within castes. Empirical studies have shown that caste, as well as task specialization within castes, is partly genetically determined (reviewed in Oldroyd & Fewell, 2007; Schwander *et al*., 2010). Therefore, low genetic diversity might reduce colony fitness by disrupting proper division of labour. In addition to the challenges posed by high relatedness, eusocial species are also challenged by the decrease in effective population size due to extreme reproductive skew (Kent & Zayed, 2013). As a result, strong linkage disequilibrium and an increased frequency of deleterious mutations might lead to Hill–Robertson interference (Hill & Robertson, 1968), reducing the efficiency of natural selection. Several authors have suggested that eusocial species may be selected to increase recombination rates in responses to these challenges (Schmid-Hempel, 1998; Gadau *et al*., 2000, 2012; Wilfert *et al*., 2007; Sirviö *et al*., 2011; Kent & Zayed, 2013). Increased recombination could increase genotypic diversity within colonies, thereby helping eusocial societies to resist parasites and maintain proper division of labour (Oldroyd & Fewell, 2007; Wilfert *et al*., 2007). Increased recombination rates also increase the efficiency of selection – counteracting the effects of small effective population sizes that are built into eusocial societies (Kent & Zayed, 2013).

Recent analyses comparing molecular estimates of recombination between eusocial and solitary Hymenoptera found that recombination rates of eusocial Hymenoptera are indeed higher than in solitary hymenopterans or any other metazoan that has been measured (Wilfert *et al*., 2007; Sirviö *et al*., 2011). Unfortunately, these analyses were based on just a small number of taxa (six eusocial and four solitary Hymenoptera) and did not control for phylogenetic nonindependence. Measuring recombination rates is challenging and labour intensive (Stumpf & McVean, 2003; Smukowski & Noor, 2011). Therefore, it is unlikely that, in the near future, sufficient data will be available for rigorous tests of the theoretical impacts of eusocial systems on recombination rates. On the other hand, currently, it is possible to examine the relationship between eusociality and a factor that is known to be correlated with recombination rate: chromosome number. In fact, most earlier theory on recombination rate evolution in eusocial species was based on comparisons between chromosome numbers in eusocial and solitary Hymenoptera species (Sherman, 1979; Seger, 1983). As mentioned above, genetic variation is a function of the independent assortment of chromosomes and the number of crossing over events between chromosomes. Increases in the number of chromosomes increase the possible genotypes due to independent assortment during meiosis. Because the number of crossing-over events is roughly constant (1–2 chiasmata), per chromosome (White, 1973) increases in chromosome number also affects recombination rate by increasing the total number of crossover events.

Here, we perform phylogenetically controlled analyses to compare chromosome numbers among eusocial and solitary species. Although the theory on recombination rates in Hymenoptera was developed to explain absolute differences in chromosome number, it can also be used to predict differences in the variance in chromosome number between solitary and eusocial species. Eusocial species vary in terms of their size, mating systems and social complexity. Each of these factors is expected to be important in shaping the evolution of recombination rates. Therefore, we predict that the variance in recombination rates – as evidenced by chromosome number – will be greater in eusocial species. As previously mentioned, the size of eusocial societies ranges from a few to millions of individuals. Kent & Zayed (2013) predicted that larger colonies are under stronger selection to increase recombination, due to an increase in reproductive skew. Larger colonies are also more likely to suffer from parasites (Schmid-Hempel, 1998) and be faced with the maintenance of more elaborate caste structures than smaller ones (Oldroyd & Fewell, 2007). We therefore expect larger colonies to benefit more from increased genotypic diversity. Indeed, Schmid-Hempel (1998) found a positive relationship between chromosome number and colonies size across 58 ant species. Recombination rates might also be affected by other factors that vary among eusocial sociaties: e.g., polyandry (multiple mating) and polygyny (multiple queens per nest). Both reduce reproductive skew, thereby increasing effective population size and decreasing selection on recombination. They also increase genotypic variation within colonies and might therefore lead to less stringent selection to increase recombination. Finally, eusocial taxa differ in their social complexity. Eusociality can be facultative, or obligate, and among eusocial species, the number of distinct castes varies. It is currently unclear how the differences between facultative and obligate eusociality might affect selection on recombination rates. However, the theory on genetic caste determination predicts that species with more castes should benefit more from increased genotypic diversity (Oldroyd & Fewell, 2007). Furthermore, colonies with many distinct castes might be selected to increase recombination as they could benefit from breaking up linkage between genes that are selected in opposite directions in different castes (Kent & Zayed, 2013).

In summary, we expect eusocial species to have a higher variance in chromosome number than solitary species, because eusocial species are variable for a number of life history parameters that are expected to affect recombination rate evolution and that are not applicable to solitary species. We test this prediction by comparing the rates of chromosome number evolution between solitary and eusocial species, as well as by explicitly modelling those factors that we expect to affect chromosome number: colony size, caste number, degree of sociality and colony relatedness (polyandry and polygyny). An alternative explanation for a high variance in chromosome number among eusocial species is that it is due to genetic drift. Changes in chromosome number are often slightly deleterious (Max, 1995), and if effective population size is reduced in eusocial lineages, chromosome number should change more quickly than in solitary species. We aim to distinguish between drift and the adaptive explanations described above by considering the effect of effective population size (assessed as the geographic range of a species) on chromosome numbers in the ants. Finally, we consider chromosome number of social parasites; as these species have lost their worker caste, there is less reproductive skew and no need to increase colony genotypic diversity and we would therefore expect them to have lower chromosome numbers than their eusocial relatives (Wilfert *et al*., 2007; Kent & Zayed, 2013).

## Methods

### Data collection

The data used for this analysis were collected from the literature between January 2012 and September 2013. We used a variety of sources, including the primary literature as well as a number of key review papers (see Table S1 and S2). References were identified via Web of Science and Google Scholar as well as by inspecting the references of all papers of interest and by searching for citations of key papers. We collected all karyotype data that have been published for species of Hymenoptera; these data are available from the Tree of Sex Database (Tree of Sex Consortium, 2014). For each species with karyotype data, we recorded eusocial status according to the definition by (Crespi & Yanega, 1995) (solitary, cooperative breeder, facultatively eusocial, obligately eusocial, with the latter two being considered ‘eusocial’), and for each eusocial species, we recorded colony size, queen number and mating numbers for single queens. Genus-level caste number estimate for ants was based on data collected by Oster & Wilson (1978). Several ant species are social parasites – they do not produce any workers themselves, but rely on those of other species. We obtained social parasite status from a recent review (Buschinger, 2009), restricting social parasites to those species with either dulosis or inquilinism. In total, we have data for 1567 species, although the character matrix is not complete for every species. We provide all data including references in Table S1 and S2. Finally, to estimate the geographic range of the ants for which we have karyotype data, we wrote a Python script to gather all of the geospatial specimen data available from AntWeb (http://www.antweb.org/). Then, using 0.5 degree grid cells, we calculated area of occupancy scores (Gaston, 1996) for each ant species. Area of occupancy score calculations were automated with another Python script.

### Phylogeny

We estimated time-scaled phylogenetic relationships among Hymenoptera lineages using a phyloinformatic approach. We used a Python script to download published DNA sequences from the NCBI nucleotide database (GenBank) which were sampled from species included in our trait data set. We targeted nine phylogenetic markers that have been used extensively in Hymenoptera phylogenetics: CAD, abdominal A, arginine kinase, elongation factor 1-alpha, long-wavelength rhodopsin, wingless, COI, cytB and the mitochondrial large ribosomal subunit 16S. GenBank accession numbers are provided in supplemental Table S2. We aligned sequence clusters with MAFFT (Katoh & Toh, 2008) and pushed the 16S alignment through Gblocks (Talavera & Castresana, 2007) to remove hypervariable regions. We used Mesquite v2.75 (Maddison & Maddison, 2013) to concatenate alignments, delete introns and delimit codon positions. The final data matrix dimensions were 602 taxa by 5600 aligned sites. We estimated phylogenetic relationships and divergence times simultaneously using BEAST v1.7.5 (Drummond & Rambaut, 2007). In the BEAST analysis, we estimated nucleotide substitution model parameters independently across five partitions: nuclear codon positions 1 + 2, nuclear codon position 3, mitochondrial codon positions 1 + 2, mitochondrial codon position 3 and the mitochondrial ribosomal positions. We assumed an HKY site model with among-site rate variation modelled with a gamma distribution, a birth–death model of phylogenetic branching, and a log-normal relaxed clock model of among-lineage substitution rate variation. We calibrated divergence time estimates with three exponential node priors: (1) 185 Ma on the stem node of Ichneumonoidea (Zessin, 1981), (2) 180 Ma on the crown node of Tenthredinoidea (Geinitz, 1887; Nel *et al*., 2004) and (3) 197 Ma on the crown node of Vespomorpha (Heer, 1865). We ran the BEAST analysis for 50 million iterations, discarding the first 42 million iterations before the stationary distribution of parameter values was reached.

### Sister-group comparison

We compared the average chromosome number of species in eusocial clades to the average chromosome numbers in their solitary sister groups. Eusocial/solitary sister groups were derived from recently published molecular phylogenies (Table S1 for references). Significance of the contrasts was evaluated with Siegel's randomization test for matched pairs (Hardy & Cook, 2010). Sister-group comparisons provide a simple and conservative test of the effect of eusociality on chromosome number. Because they require only the most basic knowledge of phylogenetic relationships, we were able to include more of the origins of eusociality in these comparisons than in our other, more parametric analyses.

### Comparative analysis

For the analysis of chromosome number evolution, we used haploid chromosome count. In cases where more than one record was available, we used the mean of all records. We analysed the data using a phylogenetic and taxonomic mixed model approach (Hadfield & Nakagawa, 2010) in the R package MCMCglmm (Hadfield, 2010a), assuming a Brownian model for the phylogenetic or taxonomic effects (Hadfield & Nakagawa, 2010). We corrected for phylogenetic nonindependence using either nested taxonomy (Superfamily/Family/subfamily/Genus) or the reconstructed molecular phylogeny described above. For all MCMCglmm analyses, we used mixed models with a Gaussian error structure and log-transformed haploid chromosome number as the response variable. As predictors, we included eusociality (solitary vs. eusocial) as a binary trait, or degree of sociality (0 = solitary, 1 = cooperative breeder, 2 = facultative eusocial, 3 = obligate eusocial) as either categorical or continuous. For the analyses of chromosome numbers within the eusocial Hymenoptera, we included colony size (log-transformed) as a continuous variable and considered both a linear and polynomial model. We combined our data on polygyny and polyandry into one binary variable ‘relatedness’: species with one or the other (or both) were scored as ‘low relatedness’, whereas singly mated monogynous colonies were scored as ‘high relatedness’. This decision was based on a previously observed negative correlation between the two (Hughes *et al*., 2008). We used inverse-gamma priors for the residual variance and parameter-expanded priors for the random effects (Hadfield, 2010b). We provided our R code for our prior specification in the Appendix 1. All models were run for 13 million iterations with a burn-in of 3 million iterations. We report the significance of our fixed effects in terms of pMCMC, which is twice the posterior probability that the estimate is negative or positive (whichever probability is smallest). This value can be interpreted as a Bayesian equivalent to the traditional *P*-value (Hadfield, 2010a; Hadfield *et al*., 2013). Finally, we validated our MCMCglmm analysis of the effect of eusociality on mean chromosome number using the R package Phytools version 0.3–72 (Revell, 2011). We conducted a phylogenetically corrected one-way anova (sensu Garland *et al*., 1993) comparing eusocial and solitary Hymenoptera. *P*-values were calculated based on a null distribution generated from 1000 simulations.

In addition to differences in mean chromosome number, eusociality might also affect the rate at which chromosome number evolves. We tested for a shift in the rate of chromosome number evolution using a censored rate test, based on a Brownian motion model. This allows us to compare models where the continuous trait (chromosome number) evolves at a single rate on all branches to a model where each state (e.g. solitary and eusocial) has an independent rate of evolution (O'Meara *et al*., 2006). Conducting the censored rate test requires a reconstruction of the history of eusociality on our tree. As eusociality is widely accepted as a derived state within Hymenoptera, we fixed the root state of the tree as solitary (Wilson, 1975). We used an MK (a continuous-time Markov chain) model to estimate the parameters of the transition rate matrix and allowed different transition rates between states. Stochastic mapping was used to assign the state of all branches in the tree. To account for uncertainty in ancestral states, we performed our analysis across 100 stochastically mapped trees. This analysis was repeated coding social state into four categories (solitary, cooperative breeder, facultatively eusocial and obligately eusocial) and two categories (solitary and eusocial). The R package Phytools version 0.3–72 was used to both reconstruct ancestral states and fit models of chromosome number evolution. Tests were considered significant at *α *= 0.05.

## Results

We present results from three types of analyses: sister-group comparisons, taxonomic mixed model estimation and a number of formal phylogenetic comparative approaches. In the taxonomic mixed model, we used a nested taxonomy to correct for shared ancestry, and our estimates were based on all 1567 species in the trait data set. The phylogeny-based approaches included only those species for which we have phylogenetic data (602 spp.). Tree-based analyses are considered the best way to control for phylogenetic nonindependence (Felsenstein, 1985; Hadfield & Nakagawa, 2010). We have included the sister-group and taxonomic analyses because it allows us to maximize the number of origins of eusociality captured by our analysis and compare these with results from tree-based approaches.

### Sister-group comparisons

In the most recent reconstruction, eusociality was estimated to have evolved nine times, and lost once in Hymenoptera (Table1 and references therein). We compared the average chromosome number of each of seven eusocial clades with that of their solitary sister group (see Table1). We lacked sufficient data for the other three eusocial clades. We found that in four of these comparisons eusocial species have more chromosomes. When we excluded those comparisons for which we have little data (by setting an arbitrary minimum number of four species for each clade), we were only left with three comparisons, all of which have higher number of chromosomes in eusocial taxa. However, due to the low replication, the contrast was nonsignificant (*P*-value = 0.25). We present results from more powerful mixed model approaches next.

**Table 1:** Sister group comparison between eusocial clades and their solitary sister groups

Superfamily	Family	Contrast	Sister groups	Eusocial	Chom.num	*N*	Origin/loss	Phyl ref	Support
Apoidea	Apidae	1	Euglossini	0	19.25	4	Loss	Danforth *et al*. 2013	−
Apoidea	Apidae	1	Meliponini + Apini + Bombini	1	16.17	153		Danforth *et al*. 2013	
Apoidea	Apidae	2	Meliponini + Apini + Bombini (-Euglossini)	1	16.17	153	Origin	Danforth *et al*. 2013	+
Apoidea	Apidae	2	Eucerini + Emphorini + Exomalopsini	0	11.80	5		Danforth *et al*. 2013	
Apoidea	Apidae	3	Allodapini	1	NA	0	Origin	Danforth *et al*. 2013	?
Apoidea	Crabronidae	4	Microstigmus	1	3.75	4	Origin	Danforth *et al*. 2013	?
Apoidea	Crabronidae	4	Nonsocial Crabronidae	0	NA	0		Danforth *et al*. 2013	
Apoidea	Halictidae	5	Halictus + Lasioglossum	1	13.50	8	Origin	Danforth *et al*. 2013	−
Apoidea	Halictidae	5	Agapostemon	0	17.00	1		Danforth *et al*. 2013	
Apoidea	Halictidae	6	Augochlorini (eusocial)	1	16.00	1	Origin	Danforth *et al*. 2013	+
Apoidea	Halictidae	6	Augochlorini (solitary)	0	11.67	3		Danforth *et al*. 2013	
‘Vespoidea’	Formicidae	7	Formicidae	1	16.29	793	Origin	Johnson *et al*. 2013	+
Apoidea	Apoidea	7	Apoidea (all nonsocial)	0	14.48	48		Johnson *et al*. 2013	
Vespoidea	Vespidae	8	Stenogastrinae	1	7.00	1	Origin	Hines *et al*. 2007	−
Vespoidea	Vespidae	8	Eumeninae (-Polistinae + Vespinae)	0	7.78	9		Hines *et al*. 2007	
Vespoidea	Vespidae	9	Polistinae + Vespinae	1	24.48	26	Origin	Hines *et al*. 2007	+
Vespoidea	Vespidae	9	Eumeninae	0	7.78	9		Hines *et al*. 2007	

### Eusociality-depended differences in chromosome number

**Figure 1:**
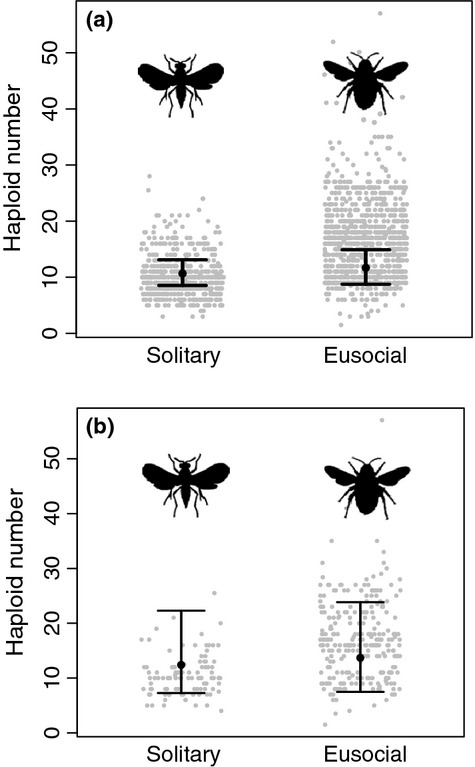
Chromosome numbers and model estimate for solitary and eusocial taxa. Each graph shows the haploid chromosome numbers for solitary and eusocial species, as well as the MCMCglmm (Hadfield, 2010a,b) posterior modes with their 95% credibility interval as error bars (calculated using the ‘summary’ function in MCMCglmm). (a) Chromosome numbers for all solitary and eusocial species for which data are available. Shown with estimates from a taxonomic mixed model in MCMCglmm. (b) Chromosome number of solitary and eusocial species for which we have phylogenetic data with model estimates from a phylogenetic mixed model in MCMCglmm. Comparing the rough data with the model estimates, it is clear why earlier analyses, which did not control for phylogenetic nonindependence, observed a clear difference between solitary and eusocial species. In fact, much of this difference is driven by the difference between the Aculeata (which consist of both solitary and eusocial species and has *n* = 16 chromosomes on average), and the rest of the Hymenoptera (with an average of *n* = 10 chromosomes) and so the effect disappears when you correct for phylogeny.

Figure1a shows a plot of the raw data on chromosome number among the Hymenoptera based on 1567 species. When we analysed this data using a taxonomic mixed model, we found that eusocial species have on average *n* = 1.1 (CI: 0.91–1.29) more chromosomes than solitary species, but this difference is not significant (p_MCMC _= 0.31). The phylogeny-based comparative analyses were based on data from 367 species (as not all species we have chromosome data for are represented in the phylogeny). We used two different methods: the phylogenetic mixed model (Hadfield & Nakagawa, 2010) and a phylogenetically corrected one-way anova. In the phylogenetic mixed model estimate, eusocial species were found to have on average *n* = 1.11 (CI: 0.77–1.59, Fig.1b, Table S3) more chromosomes than solitary ones, but the variance associated with this estimate was broad, and therefore was not significantly different from zero (p_MCMC _= 0.61). These models also show that the phylogenetic signal – akin to Pagel's lambda – of chromosome number is high (*λ *= 0.98, CI: 0.95–0.99), which means that closely related species have a high probability of sharing similar chromosome number. The phylogenetically corrected one-way anova comparing eusocial and solitary Hymenoptera also revealed no significant difference in chromosome number (*F*-statistic = 70.5, *P*-value = 0.23). We also compared chromosome numbers between solitary species, cooperative breeders, and facultatively and obligately eusocial species, but found no significant effects of the ‘degree of sociality’ (p_MCMC _= 0.804, Table S4).

### Rates of chromosome number evolution

**Figure 2:**
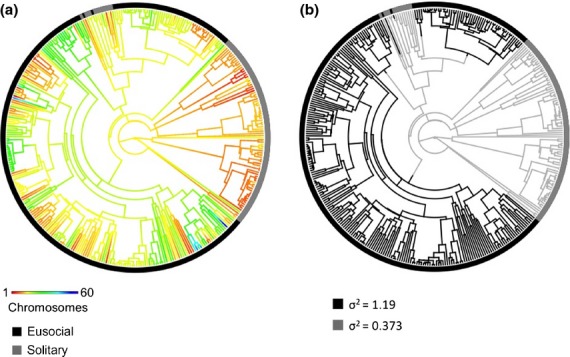
Analysis of chromosome number evolution rate. In both trees, the ring outside of the tree indicates the sociality state of the terminal taxa, black indicating eusocial and grey indicating solitary. (a) The branches have been painted to illustrate a reconstruction of the evolution of chromosome number under Brownian motion according to the scale at lower left. (b) A single stochastic mapping from our analysis shows how the branches were assigned to either eusocial or solitary states. Values for *σ*^2^ are the mean across 100 stochastic mappings and represent the rate of chromosome number evolution.

Karyotypes vary dramatically within and among the eusocial clades, with chromosome number ranging from *n* = 1 in the ant *Myrmecia croslandi* to *n* = 53–60 in the ant *Dinoponera lucida*. Here, we test whether this variation could be due to an increased rate of evolution of karyotypes in eusocial compared to solitary clades. Figure2a shows a reconstruction of Hymenoptera chromosome number evolution under Brownian motion. The censored rate test supports the notion that chromosome number evolves more quickly in eusocial than in solitary Hymenoptera. We performed the censored rate test coding taxa into four social states: solitary, cooperative breeder, facultatively eusocial and obligately eusocial. A four-rate model where each social state has its own rate was preferred across all stochastic mappings. The rate in the eusocial (facultative and obligate) clades was ∼3x faster than the solitary clades (1.28 and 1.10 vs. 0.374, respectively). We estimated the highest rate (7.69) in the cooperative breeder clade, but this group is only represented by four species on the tree, and the 95% confidence interval (2.01–13.37) indicated insufficient data to reliably estimate this rate. We repeated this analysis coding taxa as eusocial or solitary. Using this coding, we again found support for the more complex model where each social state has its own rate of evolution. This two-rate model was preferred across all 100 stochastic mappings of eusociality with a *P*-value of less than 0.001. The rate of chromosome number evolution in eusocial clades was again ∼ 3x faster (3.17–3.25x faster across stochastic character mappings) than in solitary clades (Fig.2b).

### Explaining variation within eusocial taxa

**Figure 3:**
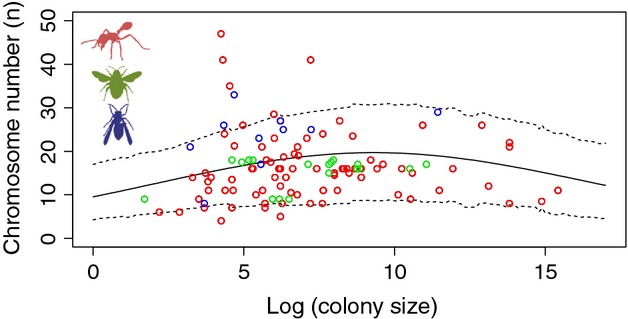
The correlation between chromosome number and colony size. The open dots show the raw data, whereas the solid lines show the model estimation from a phylogenetic mixed model in MCMCglmm generated using the ‘predict’ function. The dotted lines show the 95% credibility interval around the estimates. Data from different taxonomic groups are colour-coded (ants = red, bees = green and wasp = blue).

Colony size varies across six orders of magnitude among the eusocial Hymenoptera. Theories for the evolution of recombination among eusocial species predict a positive relationship between recombination rate and colony size. We performed a phylogenetic mixed model analysis to test that prediction. When just estimating a linear term, there was no significant effect (p_MCMC = _0.324, Table S5). However, we did estimate a significant positive nonlinear relationship between the two (p_MCMC _< 0.001 for both the first- and the second-order polynomial, see Fig.3 and Table S5), suggesting that chromosome numbers are highest in intermediate-sized colonies. We repeated this last analysis only including obligately eusocial species and found the same result (Table S5).

Next, we examined the effect of polygyny or polyandry (combined into the binary variable ‘relatedness’). We predicted that both would reduce selection for increased recombination rates.

We found that low-relatedness colonies indeed have less chromosomes (*n* = 1), but that this difference is not significant p_MCMC = _0.37. We also considered whether there is a different relationship between chromosome number and colony size for high- and low-relatedness colonies. We narrowed down our data to only those species for which we have both colony size and relatedness data (*n* = 85). We found that chromosome number positively correlates with colony size (p_MCMC _< 0.001 for both the first- and the second-order polynomial), but that neither the intercept (p_MCMC = _0.366), nor the slope (p_MCMC = _0.22) differs significantly between low- and high-relatedness colonies (Table S6).

**Figure 4:**
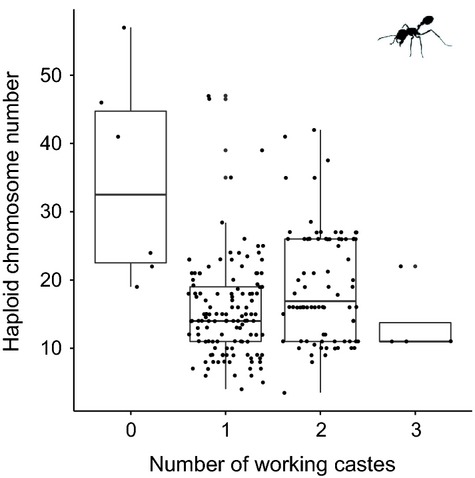
Chromosome numbers of species with varying numbers of worker castes. Worker caste number is defined as the following: 0 = no morphological difference between worker and reproductive castes, 1 = morphologically different worker and reproductive castes, but no within-worker caste polymorphism, 2 = two separate morphologically distinct worker castes. 3 = three or more morphologically distinct workers castes. The boxplot shows the interquartile range that contains values between 25th and 75th percentile. The line inside the box shows the median. The two ‘whiskers’ show the largest/smallest observation that is less than or equal to the upper quartile plus/minus 1.5 the length of the interquartile range.

Finally, we considered the relationship between caste number and chromosome number in ants (as this is where it varies the most and where most data are available). Most eusocial hymenopterans have two morphologically distinct castes: the workers and the reproductives. However, in some species, particularly ants, multiple worker castes have evolved, and in others, the morphological difference between queens and workers has been lost. Here, we combine genus-level estimates of caste number in ants with our data on chromosome number. Based on 215 species, we show that chromosome numbers indeed differ significantly among ants with different caste number (Table S7), although much of this difference is driven by the higher chromosome number in species without morphological difference between queens and workers and not by differences between species with a varying number of worker castes (Fig.4).

### Loss of eusociality

**Figure 5:**
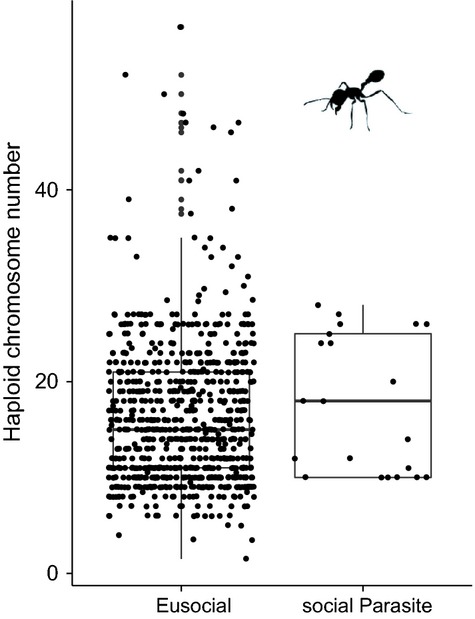
Chromosome number comparison between social parasites and their eusocial relatives. Haploid chromosome numbers. We only consider those social parasites that completely lack their own worker caste. The boxplot shows the interquartile range that contains values between 25th and 75th percentile. The line inside the box shows the median. The two ‘whiskers’ show the largest/smallest observation that is less than or equal to the upper quartile plus/minus 1.5 the length of the interquartile range.

If eusociality poses a selection pressure to increase chromosome number, we might expect that when eusociality is lost, this leads to a reduction of chromosome number. Transitions from eusociality to a solitary or subsocial lifestyle are rare. A recent molecular phylogeny of bees suggests that this transition might have taken places once among the eusocial Hymenoptera in the Euglossini (although this is somewhat controversial). Comparing the Euglossini with their eusocial sister group (Table1), the hypothesis that the loss of eusociality should be accompanied by a decrease in chromosome number is not supported, as the Euglossini have a higher average chromosome number than their sister group. Most of the putative losses of eusociality are associated with the evolution of social parasites, that is, species that do not produce their own workers, but rely on those of other species. Focusing on ants, as this is where the best data are available, we compare the chromosome number of eusocial species with those of social parasites, using a taxonomic mixed model. Contrary to our expectation, we find that social parasites on average have *n* = 4.04 (CI: 0.74–7.17) more chromosomes than eusocial species (p_MCMC _= 0.02, see Fig.5). It is not clear why this is the case, although strong antagonistic coevolution between social parasites and their hosts might have selected for an increase in recombination.

### Chromosome number and geographic range

**Figure 6:**
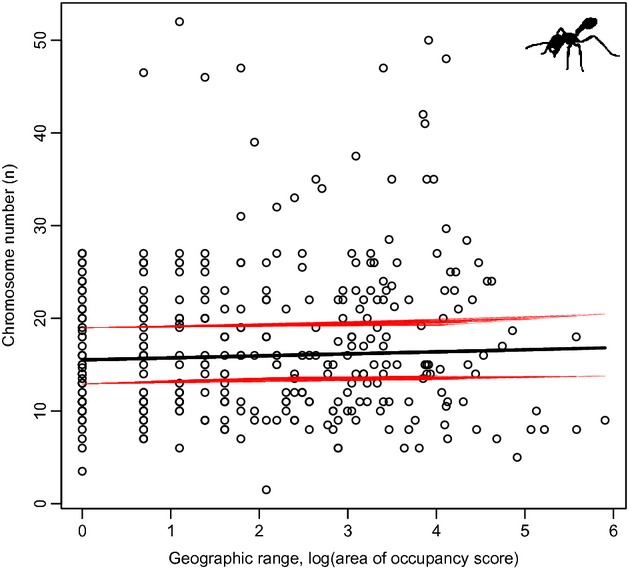
Chromosome number and geographic distribution in ants. The open dots show the raw data, whereas the solid lines show the model estimation from a phylogenetic mixed model in MCMCglmm generated using the ‘predict’ function. The red lines show the 95% credibility interval around the estimates.

Eusociality reduces the effective population size of a species through reproductive skew. This might lead to selection to increase recombination to alleviate negative effects of small effective population sizes and could explain some of the variation in chromosome number that we described previously. Effective population size, however, is also affected by the geographic range of a species and its density within that range. We therefore estimate the correlation between chromosome number and geographic range in ants. Based on estimates from 338 species, we found that there is no such relationship (p_MCMC _= 0.77, see Fig.6).

## Discussion

The observation of extraordinarily high rates of recombination in a number of eusocial Hymenoptera has spurred the development of theories explaining this pattern. Broadly speaking, these theories fall into two classes: Either eusociality leads to selection for high recombination rates to increase genotypic diversity within a colony (‘the genetic diversity theory’ Wilfert *et al*., 2007), or alternatively to alleviate the negative effects of reproductive skew and small effective population size (‘the reproductive skew theory’, Kent & Zayed, 2013). Although the theoretical validity of these models is clear, the empirical support for a causal link between eusociality and high recombination remains weak. In this study, we test the hypothesis that eusocial behaviour has affected selection on recombination rates in the Hymenoptera, using chromosome number as a proxy for recombination. Using both a taxonomic and phylogenetic framework, we show that, in contrast to earlier studies (Sherman, 1979; Seger, 1983; Wilfert *et al*., 2007), there is little support for a higher number of chromosomes among eusocial species. We believe that this is unlikely to be an issue of statistical power, as our study is the most comprehensive to date and captures four (in a phylogenetic framework) to eight (in a taxonomic framework) of the nine origins of eusociality. A more likely explanation is that the differences in chromosome number/recombination rate found by earlier analyses were due to the phylogenetic nonindependence of observations; the principal difference observed was between the Aculeata (which consist of both solitary and eusocial species and has *n* = 16 chromosomes on average) and the rest of the Hymenoptera (with an average of *n* = 10 chromosomes).

Eusocial societies are diverse. Theory predicts that selection for increased recombination rates depends on many aspects a species' ecology (Wilfert *et al*., 2007; Sirviö *et al*., 2011; Kent & Zayed, 2013). We therefore decided not just to test the absolute difference between the chromosome number of solitary and eusocial species, but also to consider the rate at which chromosome number has evolved. Using a phylogenetic framework, we found that chromosome number indeed changes at a higher rate in eusocial hymenopterans.

Next, we considered what aspects of eusocial species' ecology could explain this higher rate of evolution. Both the ‘genetic diversity’ as well as the ‘reproductive skew’ theory predicts higher rates in larger colonies. Indeed, Schmid-Hempel (1998) found a positive relationship between chromosome number and colony size across 58 ant species. We repeated Schmid-Hempel's (1998) test in a phylogenetic framework and with data across multiple origins of eusociality. We found that there is a relationship, but that it is more complex than initially thought, with the highest chromosome numbers in intermediate-sized colonies. This pattern could be explained by species with the largest colonies evolving alternative ways of increasing genetic diversity or because such species are so successful that they tend to have high effective population sizes despite their reproductive skew.

To better understand the relationship between chromosome number and colony size, we considered it separately for colonies that have polygyny or polyandry (low relatedness) or those with a single, single-mated queen (high relatedness). If genotypic diversity is what drives the relationship between colony size and chromosome number, it should be less pronounced in polygynous and polyandrous colonies, as they have other means of increasing genotypic diversity. We found that on average high-relatedness colonies had indeed a slightly higher number of chromosomes for a given colony size, but that this difference was not statistically significant. It is unclear whether this is due to a lack of statistical power (*n* = 83 for this analysis), or because there is simply no effect.

Although most of our analysis was focused on explaining the variance in chromosome number among eusocial societies, we also considered what would happen in those cases where eusociality was lost and species reverted back to either a solitary, cooperative breeding or social parasitic lifestyle. To our surprise, in three separate analyses, we found that species that secondarily lost eusociality have on average a higher number of chromosomes: this is true for the Euglossini in comparison with their eusocial sister group (Table1), for colonies that have lost a morphologically distinct reproductive caste (gamergate colonies, Fig.4) and for species that have lost their own worker caste and rely instead on those of other species (Fig.5). Due to the rarity of loss, each of these analyses is based on a relatively small sample of species, and by itself may not be convincing. However, together, they paint the picture that loss of eusocial behaviour tends to be accompanied by a rise in chromosome number. Why? This is currently unclear; it is possible that the transition to a new lifestyle is often accompanied by a bottleneck in population size and that the changes we observe are due to drift, or that the transition requires an elevated rate of adaptation to changing conditions. This might particularly apply to social parasites, where red-queen dynamics (Jaenike, 1978; Ladle, 1992) caused by antagonistic coevolution between social parasites and their hosts could select for high recombination rates.

The two theories for patterns of recombination across eusocial species are based either on its effect on genotypic diversity within colonies, or on its effect on linkage disequilibrium and interference. Although these theories are far from mutually exclusive, can we identify which of them is likely to explain most of the variation we observe? In general, both theories make very similar predictions. However, the former theory exclusively focuses on patterns of genetic variation within colonies, whereas the latter is also concerned with population-level genetic diversity (through *N*_e_). Although *N*_e_ is affected by the reproductive skew in populations, it is also affected by distribution and density of a population as a whole. This leads to the prediction of a positive correlation between the scarcity of a species and its recombination rate. It is extremely challenging to obtain census population size estimates for many species, but we were able to obtain geographic range data for most species of ants for which we have estimates of chromosome number. We were unable though to detect a correlation between the two. This suggests that either effective population size is not a strong determinant of recombination rate, or that our measure of geographic range is not a good enough predictor of *N*_e_ to detect such effect.

Much of the published theory for recombination rate and its effect on genetic variation was co-opted from theories for the evolution of polyandry. There is a wealth of empirical support for the importance of polyandry on within-colony diversity. However, it is unclear whether these theories make the same predictions about recombination. From the quantitative genetics literature, we know that additive genetic variance *decreases* with recombination rate (Falconer & Mackay, 1996). Another issue is that for example parasite resistance could be due to a single locus, in which case recombination rate would have no effect on the distribution of resistance among colony members. The same would be true if the trait is polygenic, but the effects of each gene are completely additive (Falconer & Mackay, 1996). A recent simulation study investigated the role of chromosome number and recombination rate on polygenic traits (Rueppell *et al*., 2012). They showed that both processes in general do not affect genetic variance, but that they can increase the number of unique genotypes and the genotypic range in a colony (Rueppell *et al*., 2012). However, the effects are small, and we currently know too little about the genetic architecture of traits determining colony fitness to access the impacts of these effects.

In this study, we used chromosome number as a proxy for recombination rate. This means that although we were able to utilize data from a larger sample of eusocial species, we were working with only a coarse approximation of recombination rate. For example, it is possible that recombination rate varies by means other than chromosome number, for example an increase in the number of chiasmata per chromosome. Unfortunately, most karyotypic studies of the Hymenoptera do not cite the number of chiasmata (Gokhman, 2009). However, Hymenoptera have relatively small chromosomes and it is therefore unlikely that more than one chiasmata is present per arm.

In conclusion, a theoretical link has been made between high rates of recombination, high levels of genetic diversity, effective population size and colony performance in eusocial species. Higher genotypic diversity within colonies could improve their performance, by increasing resistance to parasites, and/or maintaining the genetic basis of the division of labour. At the same time high recombination rates can reduce linkage and interference effects resulting from high reproductive skew. In this study, we tested these predictions using a large comparative analysis of Hymenoptera that assumed recombination rate is correlated with chromosome number. We found that eusociality is associated with an increased rate of chromosome number evolution and that among eusocial lineages, differences in chromosome number can be explained in part by colony size. However, the effects of a number of other ecological factors predicted to influence chromosome number – including colony complexity and relatedness – were equivocal at best. Whereas the higher rate of chromosome number evolution observed in eusocial clades may indicate varying strengths of selection on chromosome number, it may also be due to drift. Changes in chromosome number are often slightly deleterious (Max, 1995). Therefore, if effective population size is reduced in eusocial lineages, chromosome number should change more quickly due to drift. However, this is based on the assumption that eusociality always reduces effective population size, which fails to take into account the ecological success of many eusocial species. We show that at least in ants, more widely distributed species (that presumably have a higher effective population size) do not show a reduction in chromosome number variability (Fig.6), suggesting that drift is not the main driver of chromosome number evolution. But of course, this analysis is not conclusive: historical population size is the most important parameter affecting effective population size, and it is unclear how historical population size correlates with current distribution. The current theory on recombination rate evolution in eusocial invertebrates fails to evaluate the relative importance of linkage disequilibrium and colony genotypic diversity. As a result it fails to provide clear quantitative predictions for both short- and long-term effects of recombination rate. Such theoretical advances, in addition to recombination rate estimates for more Hymenoptera species, especially solitary members of the Aculeata, gamergate colonies and social parasites, will be crucial.

## APPENDIX

### Appendix 1 Priors used for all MCMCglmm analyses

For all models, we used an inverse-gamma with shape = scale = 0.001 for the residual variance and for the random effects a parameter-expanded prior resulting in a scaled (1000) F-distribution with 1 degree of freedom for the numerator and denominator as a prior distribution.

#### For taxonomic mixed model

prior<-list(R=list(V=1,nu=0.002),G=list(G1=list(V=1,nu=1, alpha.mu=0, alpha.V=1000), G2=list(V=1,nu=1, alpha.mu=0, alpha.V=1000),G3=list(V=1,nu=1, alpha.mu=0, alpha.V=1000),G4=list(V=1,nu=1, alpha.mu=0, alpha.V=1000)))

#### For phylogenetic mixed model

prior<-list(R=list(V=1,nu=0.002),G=list(G1=list(V=1,nu=1, alpha.mu=0, alpha.V=1000)))

## Supporting Information

Table S1 Supplementary data table.

Table S2 Reference list for the supplementary data table.

Table S3 Difference chromosome number between eusocial and solitary species. **Table S4** Difference chromosome number eusocial continuum. **Table S5** Correlation between chromosome number and colony size. **Table S6** Chromosome number, colony size and colony relatedness. **Table S7** Chromosome number and worker castes. **Table S8** Difference chromosome number between eusocial and social parasitic ants. **Table S9** Correlation between chromosome number and geographic range.

## References

[b1] Agrawal AF (2006). Evolution of sex: why do organisms shuffle their genotypes?. Curr. Biol.

[b2] Baer B, Schmid-Hempel P (1999). Experimental variation in polyandry affects parasite loads and fitness in a bumble-bee. Nature.

[b4] Bell G (1983). The Masterpiece of Nature: the Evolution and Genetics of Sexuality.

[b5] Boomsma JJ (2009). Lifetime monogamy and the evolution of eusociality. Philos. T Roy. Soc. B.

[b200] Buschinger A (2009). Social parasitism among ants: a review (Hymenoptera: Formicidae). Myrmecol. News.

[b6] Crespi BJ, Yanega D (1995). The definition of eusociality. Behav. Ecol.

[b300] Danforth BN, Cardinal S, Praz C, Almeida EA, Michez D (2013). The impact of molecular data on our understanding of bee phylogeny and evolution. Annu. Rev. Entomol.

[b7] Drummond AJ, Rambaut A (2007). BEAST: Bayesian evolutionary analysis by sampling trees. BMC Evol. Biol.

[b8] Falconer DS, Mackay TFC (1996). Introduction to Quantitative Genetics.

[b9] Felsenstein J (1985). Phylogenies and the comparative method. Am. Nat.

[b10] Gadau J, Page RE, Werren JH, Schmid-Hempel P (2000). Genome organization and social evolution in Hymenoptera. Naturwissenschaften.

[b11] Gadau J, Helmkampf M, Nygaard S, Roux J, Simola DF, Smith CR (2012). The genomic impact of 100 million years of social evolution in seven ant species. Trends Genet.

[b12] Garland T, Dickerman AW, Janis CM, Jones JA (1993). Phylogenetic analysis of covariance by computer simulation. Syst. Biol.

[b13] Gaston KJ (1996). Species-range-size distributions: patterns, mechanisms and implications. Trends Ecol. Evol.

[b14] Geinitz FE (1887). Neue Aufschlusse der Flozformation Mecklenburgs. IX Beitrag zur Geologie Mecklenburgs. IV Jura. Arch. des Vereins der Fruende nat. Meck.

[b15] Gokhman VE (2009). Karyotypes of Parasitic Hymenoptera.

[b16] Hadfield JD (2010a). MCMC methods for multi-response generalized linear mixed models: the MCMCglmm R Package. J. Stat. Softw.

[b17] Hadfield JD (2010b). http://cran.r-project.org/web/packages/MCMCglmm/vignettes/CourseNotes.pdf.

[b18] Hadfield J, Nakagawa S (2010). General quantitative genetic methods for comparative biology: phylogenies, taxonomies and multi-trait models for continuous and categorical characters. J. Evolution Biol.

[b19] Hadfield JD, Heap EA, Bayer F, Mittell EA, Crouch N (2013). Disentangling genetic and prenatal sources of familial resemblance across ontogeny in a wild passerine. Evolution.

[b20] Hardy NB, Cook LG (2010). Gall-induction in insects: evolutionary dead-end or speciation driver?. BMC Evol. Biol.

[b21] Heer O (1865). Die Urwelt der Schweitz.

[b22] Hill WG, Robertson A (1968). Linkage disequilibrium in finite populations. Theor. Appl. Genet.

[b23] Hughes WOH, Boomsma JJ (2004). Genetic diversity and disease resistance in leaf-cutting ant societies. Evolution.

[b400] Hines HM, Hunt JH, O'Connor TK, Gillespie JJ, Cameron SA (2007). Multigene phylogeny reveals eusociality evolved twice in vespid wasps. Proc. Natl. Acad. Sci. U S A.

[b24] Hughes WOH, Ratnieks FLW, Oldroyd BP (2008). Multiple paternity or multiple queens: two routes to greater intracolonial genetic diversity in the eusocial Hymenoptera. J. Evolution Biol.

[b25] Jaenike J (1978). An hypothesis to account for the maintenance of sex within populations. Evol. Theory.

[b500] Johnson BR, Borowiec ML, Chiu JC, Lee EK, Atallah J, Ward PS (2013). Phylogenomics resolves evolutionary relationships among ants, bees, and wasps. Curr. Biol.

[b26] Katoh K, Toh H (2008). Recent developments in the MAFFT multiple sequence alignment program. Brief. Bioinformat.

[b27] Kent CF, Zayed A (2013). Evolution of recombination and genome structure in eusocial insects. Commun. Integr. Biol.

[b28] Kraus B, Page RE (1998). Parasites, pathogens, and polyandry in social insects. Am. Nat.

[b29] Ladle RJ (1992). Parasites and sex: catching the red queen. Trends Ecol. Evol.

[b30] Lenormand T, Dutheil J (2005). Recombination difference between sexes: a role for haploid selection. PLoS Biol.

[b32] Maddison WP, Maddison DR (2013). http://mesquiteproject.org/mesquite/mesquite.html.

[b600] Max K (1995). Species Evolution: the Role of Chromosome Change.

[b33] Maynard-Smith J (1978). The Evolution of sex.

[b34] Nel A, Petrulevicius JF, Henrotay M (2004). New early Jurassic sawflies from Luxembourg: the oldest record of Tenthredinoidea (Hymenoptera: “Symphyta”). Acta Palaeontol. Pol.

[b35] Oldroyd BP, Fewell JH (2007). Genetic diversity promotes homeostasis in insect colonies. Trends Ecol. Evol.

[b36] O'Meara BC, Ané C, Sanderson MJ, Wainwright PC (2006). Testing for different rates of continuous trait evolution using likelihood. Evolution.

[b37] Oster GF, Wilson EO (1978). Caste and Ecology in the Social Insects.

[b38] Otto SP (2009). The evolutionary enigma of sex. Am. Nat.

[b39] Otto SP, Lenormand T (2002). Evolution of sex resolving the paradox of sex and recombination. Nat. Rev. Genet.

[b41] Revell LJ (2011). phytools: an R package for phylogenetic comparative biology (and other things). Methods Ecol. Evol.

[b42] Rueppell O, Meier S, Deutsch R (2012). Multiple mating but not recombination causes quantitative increase in offspring genetic diversity for varying genetic architectures. PLoS One.

[b43] Schmid-Hempel P (1998). Parasites in Social Insects.

[b44] Schmid-Hempel P, Crozier R (1999). Ployandry versus polygyny versus parasites. Philos. T Roy. Soc. B.

[b45] Schwander T, Lo N, Beekman M, Oldroyd BP, Keller L (2010). Nature versus nurture in social insect caste differentiation. Trends Ecol. Evol.

[b46] Seger J, Rhodin AGJ, Miyata K (1983). Conditional relatedness, recombination, and the chromosome numbers of insects.

[b47] Sherman P (1979). Insect chromosome numbers and eusociality. Am. Nat.

[b48] Shykoff JA, Schmid-Hempel P (1991). Parasites and the advantage of genetic variability within social insect colonies. P. R. Soc. B.

[b49] Sirviö A, Johnston JS, Wenseleers T, Pamilo P (2011). A high recombination rate in eusocial Hymenoptera: evidence from the common wasp *Vespula vulgaris*. BMC Genet.

[b50] Smukowski CS, Noor MAF (2011). Recombination rate variation in closely related species. Heredity.

[b51] Stumpf MPH, McVean GAT (2003). Estimating recombination rates from population-genetic data. Nat. Rev. Genet.

[b52] Talavera G, Castresana J (2007). Improvement of phylogenies after removing divergent and ambiguously aligned blocks from protein sequence alignments. Syst. Biol.

[b53] Tarpy DR (2003). Genetic diversity within honeybee colonies prevents severe infections and promotes colony growth. P. Roy. Soc. Lond. B Bio.

[b54] Tree of Sex Consortium (2014). Tree of sex: a database of sexual systems. Scientific Data.

[b55] White MJD (1973). Animal Cytology and Evolution.

[b56] Wilfert L, Gadau J, Schmid-Hempel P (2007). Variation in genomic recombination rates among animal taxa and the case of social insects. Heredity.

[b57] Wilson EO (1975). Sociobiology.

[b58] Wilson-Rich N, Spivak M, Fefferman NH, Starks PT (2009). Genetic, individual, and group facilitation of disease resistance in insect societies. Annu. Rev. Entomol.

[b59] Zessin W (1981). Ein Hymenopteraflugen aus dem oberen Lias bei Dobbertin. Zeitschrift Fuer Geologische Wissenschaften Berlin.

