# Supplementary material for: Recombination, chromosome number and eusociality in the Hymenoptera
Source: J Evol Biol. 2015 Jan 6;28(1):105–16. doi: 10.1111/jeb.12543 (PMC4328152; doi:10.1111/jeb.12543)
Supplement: Table S2 — Reference list for the supplementary data table. [file jeb0028-0105-sd2.docx]

**References data comparative analysis**

Adams, R. & Longino, J.T. 2007. Nesting biology of the arboreal fungus-growing ant Cyphomyrmex cornutus and behavioral interactions with the social-parasitic ant Megalomyrmex mondabora. *Insectes Sociaux* **54**: 136–143.

Antonialli, W.F., Jr & Giannotti, E. 2001. Nest architecture and population dynamics of the ponerine ant Ectatomma edentatum (Hymenoptera, Formicidae). *Sociobiology* **38**: 475–486.

Ayabe, T., Hoshiba, H. & Ono, M. n.d. Cytological evidence for triploid males and females in the bumblebee, Bombus terrestris. *Chromosome Res* **12**: 215–223.

Barth, A., Fernandes, A., Pompolo, S.D.G. & Costa, M.A. 2011. Occurrence of B chromosomes in Tetragonisca Latreille, 1811 (Hymenoptera, Apidae, Meliponini): A new contribution to the cytotaxonomy of the genus. *Genet Mol Biol* **34**: 77–79.

Beckers, R., Goss, S., Deneubourg, J.L. & Pasteels, J.M. 1989. Colony Size, Communication and Ant Foraging Strategy. *Psyche: A Journal of Entomology* **96**: 239–256.

Beugnon, G., Chagné, P. & Dejean, A. 2001. Colony structure and foraging behavior in the tropical formicine ant, Gigantiops destructor. *Insectes Sociaux* **48**: 347–351.

Billen, J., Thijs, B., Ito, F. & Gobin, B. 2005. The pretarsal footprint gland of the ant Amblyopone reclinata(Hymenoptera, Formicidae) and its role in nestmate recruitment. *Arthropod Struct Dev* **34**: 111–116.

Boulay, R., Hefetz, A., Cerdá, X., Devers, S., Francke, W., Twele, R., *et al.* 2007. Production of sexuals in a fission-performing ant: dual effects of queen pheromones and colony size. *Behav Ecol Sociobiol* **61**: 1531–1541.

Brito, R.M., de Almeida Caixeiro, A.P., Pompolo, S.D.G. & Azevedo, G.G. 2003. Cytogenetic data of Partamona peckolti (Hymenoptera, Apidae, Meliponini) by C banding and fluorochrome staining with DA/CMA3 and DA/DAPI. *Genet Mol Biol* **26**: 53–58.

Brito, R.M., Graças Pompolo, das, S., Magalhães, M.F.M., de Barros, E.G. & Sakamoto-Hojo, E.T. 2005. Cytogenetic characterization of two Partamona species (Hymenoptera, Apinae, Meliponini) by fluorochrome staining and localization of 18S rDNA clusters by FISH. *Cytologia* **70**: 373–380.

Brito-Ribon, R.M., Miyazawa, C.S. & Pompolo, S.G. 1999. First karyotype characterization of four species of *Partamona* (Friese, 1980) (Hymenoptera, Apidae, Meliponinae) in Mato Grosso state, Brazil. *Cytobios* **100**: 19–26.

Brown, W.D., Keller, L. & Sundström, L. 2002. Sex allocation in mound-building ants: the roles of resources and queen replenishment. *Ecology* **83**: 1945–1952.

Brown, W.L. & Ramberg, F.B. 1985. Karyotype of *Augochlora pura* (Hymenoptera: Halictidae). *Entomol. News.* **96**: 161–162.

Brunner, E. & Heinze, J. 2009. Worker dominance and policing in the ant Temnothorax unifasciatus. *Insectes Sociaux* **56**: 397–404.

Buschinger, A. 2009. Social parasitism among ants: a review (Hymenoptera: Formicidae). *Myrmecological News* **12**: 219–235.

Clement, L.W., Köppen, S.C., Brand, W.A. & Heil, M. 2008. Strategies of a parasite of the ant–Acacia mutualism. *Behav Ecol Sociobiol* **62**: 953–962.

Costa, M.A. & Melo, G.A.R. 1993. Karyotypes and heterochromatin distribution (C-band patterns) in three species of *Microstigmus* wasps (Hymenoptera, Sphecidae, Pemphredoninae). *Rev. Brasil. Genet.* **16**: 923–926.

Costa, M.A. & Miyazawa, C.S. 2004. Karyotypic description of four species of Trigona (Jurine, 1807)(Hymenoptera, Apidae, Meliponini) from the State of Mato Grosso, Brazil. *Genet Mol Biol* **27**: 187–190.

Denis, D., Pezon, A. & Fresneau, D. 2007. Reproductive allocation in multinest colonies of the ponerine ant Pachycondyla goeldii. *Ecol Entomol* **32**: 289–295.

Deodikar, G.B., Thakar, C.V. & Shah, P.N. n.d. Cyto-genetic studies in Indian honey-bees. *Proceedings of the Indian Academy of Sciences - Section B* **49**: 194–206.

Dietemann, V. 2002. Differentiation in reproductive potential and chemical communication of reproductive status in workers and queens of the ant Myrmecia gulosa. Unpublished Ph.D. thesis, Universität Würzburg, Fakultät für Biologie.

Domingues, A.M.T., Waldschmidt, A.M., Andrade, S.E., Andrade-Souza, V., Alves, R.M. de O., Silva Junior, J.C.D., *et al.* 2005. Karyotype characterization of Trigona fulviventris Guérin, 1835 (Hymenoptera, Meliponini) by C banding and fluorochrome staining: Report of a new chromosome number in the genus. *Genet Mol Biol* **28**: 390–393.

Dornhaus, A., Powell, S. & Bengston, S. 2012. Group Size and Its Effects on Collective Organization. *Annu. Rev. Entomol.* **57**: 123–141.

Eickwort, G.C. 1986. First steps into eusociality: the sweat bee Dialictus lineatulus. *Fla Entomol* 742–754.

Eltz, T., Schmid, M. & Roubik, D.W. 1997. Haploid karyotypes of two species of orchid bees (Hymenoptera: Apidae, Euglossini). *Journal of the Kansas Entomological Society* 142–144.

Fahrenhorst, H. 1977. Nachweis übereinstimmender chromosomen-zahlen. *Apidologie* **8**: 89-100.

Fjerdingstad, E.J., Gertsch, P.J. & Keller, L. 2003. The relationship between multiple mating by queens, within‐colony genetic variability and fitness in the ant Lasius niger. *J Evolution Biol* **16**: 844–853. Wiley Online Library.

Frumhoff, P.C. & Ward, P.S. 1992. Individual-level selection, colony-level selection, and the association between polygyny and worker monomorphism in ants. *Am Nat* 559–590.

Gadau, J., Helmkampf, M., Nygaard, S., Roux, J., Simola, D.F., Smith, C.R., *et al.* 2012. The genomic impact of 100 million years of social evolution in seven ant species. *Trends Genet* **28**: 14–21.

Geraghty, M.J., Dunn, R.R. & Sanders, N.J. 2007. Body size, colony size, and range size in ants (Hymenoptera: Formicidae): Are patterns along elevational and latitudinal gradients consistent with Bergmann's Rule. *Myrmecological News* **10**: 51–58.

Gobin, B., Billen, J. & Peeters, C. 2001. Dominance interactions regulate worker mating in the polygynous ponerine ant Gnamptogenys menadensis. *Ethology* **107**: 495–508.

Gokhman, V.E. 2009. *Karyotypes of parasitic Hymenoptera*. Springer.

Gomes, L.F., Brito, R.M., Pompolo, S.D.G., Campos, L.A.D.O. & Peruquetti, R.C. 1998. Karyotype and C‐and G‐Banding Patterns of Eufriesea Violacea (Hymenoptera, Apidae, Euglossinae). *Hereditas* **128**: 73–76.

Goodpasture, C. 1974. Cytological data and classification of the Hymenoptera. Unpublished Ph.D. thesis. University of California: Davis.

Gordon, D.M. 1995. The development of an ant colony's foraging range. *Animal Behaviour* **49**: 649–659.

Gotoh, A. & Ito, F. 2008. Seasonal cycle of colony structure in the Ponerine ant Pachycondyla chinensis in western Japan (Hymenoptera, Formicidae). *Insectes Sociaux* **55**: 98–104.

Gray, B. 1974. Nest structure and populations ofMyrmecia (Hymenoptera: Formicidae), with observations on the capture of prey. *Insectes Sociaux* **21**: 107–120.

Helms, K.R. 1995. Natural history of the ant Pheidole desertorum Wheeler in a desert grassland habitat. *Psyche* **102**: 35–47.

Higashi, S. & Peeters, C.P. 1990. Worker polymorphism and nest structure in Myrmecia brevinoda Forel (Hymenoptera: Formicidae). *Australian Journal of Entomology* **29**: 327–331.

Hobbs, G.A. 1965. Ecology of Species of Bombus Latr.(Hymenoptera: Apidae) in Southern Alberta. II. Subgenus Bombias Robt. *The Canadian Entomologist* **97**: 120–128.

Hora, R.R., Doums, C., Poteaux, C., Fénéron, R., Valenzuela, J., Heinze, J., *et al.* 2005. Small queens in the ant Ectatomma tuberculatum: a new case of social parasitism. *Behav Ecol Sociobiol* **59**: 285–292.

Hoshiba, H. n.d. Karyotype studies on the two species of Polistinae, *Polistes chinensis* and *P. nimpha* (Vespidae, Hymenoptera). *CIS* **40**: 26–28.

Hoshiba, H. & IMAI, H. 1993. Chromosome Evolution of Bees and Wasps (Hymenoptera, Apocrita) on the Basis of C-banding Pattern Analyses. *Japanese journal of entomology* **61**: 465–492.

Hoshiba, H. & YAMAMOTO, H. 1985. Karyological studies on the three species of the haploid males of Vespinae, Vespa mandarinia Smith, V. simillima xanthoptera Cameron and Vespula flaviceps (Smith)(Vespidae, Hymenoptera). *Proceedings of the Japan Academy. Ser. B: Physical and Biological Sciences* **61**: 67–70.

Hölldobler, B. & Wilson, E.O. 1990. *The Ants*. Harvard University Press, Harvard.

Hughes, C.R., Queller, D.C., Strassmann, J.E. & Davis, S.K. 1993. Relatedness and altruism in Polistes wasps. *Behav Ecol* **4**: 128–137.

Hughes, W.O., Oldroyd, B.P., Beekman, M. & Ratnieks, F.L. 2008. Ancestral monogamy shows kin selection is key to the evolution of eusociality. *Science* **320**: 1213–1216.

Hung, H., Reed, H.C. & Vinson, S.B. 1981. Chromosomes of four species of Polistes wasps. *Cariologica* **34**: 225–230.

Ito, F. 1994. Colony composition of two Malaysian ponerine ants, Platythyrea tricuspidata and P. quadridenta: sexual reproduction by workers and production of queens (Hymenoptera: Formicidae). *Psyche* **101**: 209–218.

Ito, F. & Higashi, S. 1990. Tests of four hypotheses on soldier production, by using wild colonies ofPheidole fervida F. Smith (Hymenoptera: Formicidae). *Res Popul Ecol* **32**: 113–117.

Ito, F. & Ohkawara, K. 2000. Production and behavior of ergatoid queens in two species of the Indonesian ponerine ant genus Leptogenys (diminuta-group)(Hymenoptera: Formicidae). *Ann Entomol Soc Am* **93**: 869–873.

Ito, F., Yusoff, N.R. & Idris, A.H. 1996. Colony composition and queen behavior in polygynous colonies of the oriental ponerine antOdontomachus rixosus (Hymenoptera Formicidae). *Insectes Sociaux* **43**: 77–86.

Jeanne, R.L. 1980. Evolution of social behavior in the Vespidae. *Annu. Rev. Entomol.* **25**: 371–396.

Junior, T., Minoru, G., Soleman, R.A. & Noll, F.B. 2010. Morphological and physiological variation between queens and workers of Protonectarina sylveirae (de Saussure)(Hymenoptera, Vespidae, Epiponini). *Revista Brasileira de Entomologia* **54**: 104–109.

Kaspari, M. & Vargo, E.L. 1995. Colony size as a buffer against seasonality: Bergmann's rule in social insects. *Am Nat* 610–632.

Kerr, W.E. 1952. A variacão do numero de cromossomos na evolução dos Hymenoptera. *Scientia Genet.* **4**: 182–190.

Kerr, W.E. 1948. Estudos sobre o gênero Melipona. *Anais da Escola Superior de Agricultura Luiz de Queiroz* **5**: 181–276.

Kerr, W.E. 1972. Numbers of chromosomes in some species of bees. *Journal of the Kansas Entomological Society* 111–122.

Kerr, W.E. 1969. *Some aspects of the evolution of social bees (Apidae)*. Appleton-Century-Crofts.

Kerr, W.E. & Araujo de, V.P. 1957. Contribuicão ao estudo citológico dos Apoidea. I. Espermatogenése em tres espécies africanas. *Gracia de Orta.* **5**: 431–433.

King, J.R. & Porter, S.D. 2007. Body size, colony size, abundance, and ecological impact of exotic ants in Florida's upland ecosystems. *Evol Ecol Res* **9**: 757.

Kirpik, M.A., Guel, S., Nur, G., Inak, S., Cilingir, M., Aldemir, A., *et al.* 2009. A study on karyotypes of two species of Anoplius (Hymenoptera: Pompilidae) in Kars Plateau, Turkey. *Kafkas Univ Vet Fak Derg* **15**: 591–593.

Krinski, D., Fernandes, A., Rocha, M.P. & Pompolo, S.D.G. 2010. Karyotypic description of the stingless bee Oxytrigona cf. flaveola (Hymenoptera, Apidae, Meliponina) of a colony from Tangará da Serra, Mato Grosso State, Brazil. *Genet Mol Biol* **33**: 494–498.

Kudô, K., Tsujita, S., Tsuchida, K., Goi, W., Yamane, S., Mateus, S., *et al.* 2005. Stable relatedness structure of the large-colony swarm-founding wasp Polybia paulista. *Behav Ecol Sociobiol* **58**: 27–35.

Kumbkarni, C.G. 1965. Cytological Studies in Hymenoptera. *Cytologia* **30**: 222–228.

Leal, I.R. & Oliveira, P.S. 1995. Behavioral ecology of the neotropical termite-hunting ant Pachycondyla (= Termitopone) marginata: colony founding, group-raiding and migratory patterns. *Behav Ecol Sociobiol* **37**: 373–383.

Liefke, C., Dorow, W., Hölldobler, B. & Maschwitz, U. 1998. Nesting and food resources of syntopic species of the ant genus Polyrhachis (Hymenoptera, Formicidae) in West-Malaysia. *Insectes Sociaux* **45**: 411–425.

Lopes, D.M., Pompolo, S.D.G., Campos, L.A.D.O. & Tavares, M.G. 2008. Cytogenetic characterization of Melipona rufiventris Lepeletier 1836 and Melipona mondury Smith 1863 (Hymenoptera, Apidae) by C banding and fluorochromes staining. *Genet Mol Biol* **31**: 49–52.

Lorite, P. & Palomeque, T. 2010. Karyotype evolution in ants (Hymenoptera: Formicidae), with a review of the known ant chromosome numbers. *Myrmecol News* **13**: 89–102.

Maffei, E.M., Pompolo, S.G., Silva-Junior, J.C. & Caixeiro, A.P. 2001. Silver staining of nucleolar organizer regions (NOR) in some species of Hymenoptera (bees and parasitic wasp) and Coleoptera (lady-beetle). *Cytobios* **104**: 119–125.

Mateus, S., Noll, F.B. & Zucchi, R. 1997. Morphological caste differences in the neotropical swarm-founding polistine wasps: Parachartergus smithii (Hymenoptera: Vespidae). *Journal of the New York Entomological Society* 129–139.

Matthews, R.W. 1968. Microstigmus comes: sociality in a sphecid wasp. *Science* **160**: 787–788.

Melo, G. & Matthews, R.W. 1997. Six new species of Microstigmus wasps (Hymenoptera: Sphecidae), with notes on their biology. *Journal of Natural History* **31**: 421–437.

Meves, F. 1907. Die Spermatocytenteilungen bei der Honigbiene (Apis meliifica L) nebst Bemerkungen über Chromatinreduktion. *Archiv für mikroskopische Anatomie* **70**: 414–491.

Michener, C.D. 1974. *The social behavior of the bees: a comparative study*. Harvard University Press.

Misra, J.S. & Srivastava, M.D.L. 1971. Chromosomal changes correlated with differentiation during embryonic development of *Polistes hebraeus* (Family: Vespidae Order: Hymenoptera). *Proc. Natl. Acad. Sci. India. Ser. B.* **41**: 97–112.

Molet, M., Van Baalen, M. & Peeters, C. 2008. Shift in Colonial Reproductive Strategy Associated with a Tropical‐Temperate Gradient in Rhytidoponera Ants. *The American Naturalist* **172**: 75–87.

Monnin, T. & Peeters, C. 2008. How many gamergates is an ant queen worth? *Naturwissenschaften* **95**: 109–116.

Murakami, T., Higashi, S. & Windsor, D. 2000. Mating frequency, colony size, polyethism and sex ratio in fungus-growing ants (Attini). *Behav Ecol Sociobiol* **48**: 276–284.

Ohkawara, K., Ito, F. & Higashi, S. 1993. Production and reproductive function of intercastes inMyrmecina gvaminicola nipponica colonies (Hymenoptera: Formicidae). *Insectes Sociaux* **40**: 1–10.

Owen, R.E. 1983. Chromosome numbers of 15 North American bumble bee species (Hymenoptera, Apidae, Bombini). *Canadian Journal of Genetics and Cytology* **25**: 26–29.

Owen, R.E., Richards, K.W. & Wilkes, A. 1995. Chromosome numbers and karyotypic variation in bumble bees (Hymenoptera: Apidae; Bombini). *Journal of the Kansas Entomological Society* 290–302.

Packer, L. & Owen, R.E. 1989. Notes on the biology of *Lasioglossum* (Evylaevs) *cooleyi* (Crawford), An eusocial Halictine bee (Hymenoptera: Halicttdae). *The Canadian Entomologist* **121**: 431–438. Cambridge Univ Press.

Pardi, L. 1947. Ricerche sui Polistini VIII. La spermatogenesis di *Polistes gallicus* (L.) e di *Polistes (Leptopolistes) omissus* (Weyrauch). *Scientia Genet.* **3**: 14–22.

Peeters, C. & Ito, F. 2001. Colony dispersal and the evolution of queen morphology in social Hymenoptera. *Annu. Rev. Entomol.* **46**: 601–630.

Peeters, C. & Molet, M. 2010. Evolution of advanced social traits in phylogenetically basal ants: striking worker polymorphism and large queens in Amblyopone australis. *Insectes Sociaux* **57**: 177–183.

Pompolo, S.D.G. & Campos, L.A.D.O. 1995. Karyotypes of two species of stingless bees, Leurotrigona muelleri and Leurotrigona pusilla (Hymenoptera, Meliponinae). *Revista Brasileira de Genética* **18**: 181–181.

Pompolo, S.D.G. & Takahashi, C.S. 1986. Karyotype of two species of wasps of the genusPolistes (Polistinae, Vespidae, Hymenoptera). *Insectes Sociaux* **33**: 142–148.

Pompolo, S.G. & Takahashi, C.S. 1990. Chromosome numbers and C-banding in two wasp species of the genusPolistes (Hymenoptera Polistine, Polistini). *Insectes Sociaux* **37**: 251–257.

Pompolo, S.G. & Takahashi, C.S. 1987. Cytogenetics of Brazilian Polybiini wasps (Hymenoptera, Vespidae, Polistinae). *Brazilian Journal of Genetics* **10**: 483–496.

Rabeling, C., Verhaagh, M. & Engels, W. 2007. Comparative study of nest architecture and colony structure of the fungus-growing ants, Mycocepurus goeldii and M. smithii. *Journal of Insect Science* **7**.

Ramberg, F.B., Kukuk, P. & Brown, W.L., Jr. 1984. Karyotypes of Three Species of Halictidae (Hymenoptera: Apoidea). *Journal of the Kansas Entomological Society* 159–161.

Raw, A. 1998. Population densities and biomass of neotropical social wasps (Hymenoptera, Vespidae) related to colony size, hunting range and wasp size. *Revista Brasileira de Zoologia* **15**: 815–822.

Rocha, M.P., Pompolo, S.D.G., DERGAM, J.A., Fernandes, A. & Campos, L.A.D.O. 2002. DNA characterization and karyotypic evolution in the bee genus Melipona (Hymenoptera, Meliponini). *Hereditas* **136**: 19–27.

Rüppell, O., HEINZE, J. & Hölldobler, B. 2001. Alternative reproductive tactics in the queen-size-dimorphic ant Leptothorax rugatulus (Emery) and their consequences for genetic population structure. *Behav Ecol Sociobiol* **50**: 189–197.

Shik, J.Z. 2008. Ant colony size and the scaling of reproductive effort. *Functional Ecology* **22**: 674–681.

Sirviö, A., Johnston, J.S., Wenseleers, T. & Pamilo, P. 2011. A high recombination rate in eusocial Hymenoptera: evidence from the common wasp Vespula vulgaris. *BMC Genet.* **12**: 95.

Tek Tay, W., Cook, J.M., Rowe, D.J. & Crozier, R.H. 1997. Migration between nests in the Australian arid‐zone ant Rhytidoponera sp. 12 revealed by DGGE analyses of mitochondrial DNA. *Mol Ecol* **6**: 403–411.

Thakar, S.S. & Deodikar, G.B. 1966a. Chromosome number in Apis florea Fab. *Curr Sci* **35**: 186.

Thakar, S.S. & Deodikar, G.B. 1966b. Chromosome number in Apis florea Fab. *Curr Sci* **35**: 186.

Thomas, M.L. & Elgar, M.A. 2003. Colony size affects division of labour in the ponerine ant Rhytidoponera metallica. *Naturwissenschaften* **90**: 88–92.

Tschinkel, W.R. 1998. Sociometry and sociogenesis of colonies of the harvester ant, Pogonomyrmex badius: worker characteristics in relation to colony size and season. *Insectes Sociaux* **45**: 385–410.

Tsuchida, K., Nagata, N. & Kojima, J. 2002. Diploid males and sex determination in a paper wasp, Polistes chinensis antennalis (Hymenoptera, Vespidae). *Insectes Sociaux* **49**: 120–124.

Tsuji, K. & Yamauchi, K. 1994. Colony level sex allocation in a polygynous and polydomous ant. *Behav Ecol Sociobiol* **34**: 157–167.

Wille, A. 1983. Biology of the stingless bees. *Annu. Rev. Entomol.* **28**: 41–64.

Yamane, S. 1996. Ecological factors influencing the colony cycle of Polistes wasps. *Natural history and evolution of paper-wasps* **75**: 97.
