# Supplementary material for: Recombination, chromosome number and eusociality in the Hymenoptera
Source: J Evol Biol. 2015 Jan 6;28(1):105–16. doi: 10.1111/jeb.12543 (PMC4328152; doi:10.1111/jeb.12543)
Supplement: Table S3 — Difference chromosome number between eusocial and solitary species. Table S4 Difference chromosome number eusocial continuum. Table S5 Correlation between chromosome number and colony size. Table S6 Chromosome number, colony size and colony relatedness. Table S7 Chromosome number and worker castes. Table S8 Difference chromosome number between eusocial and social parasitic ants. Table S9 Correlation between chromosome number and geographic range. [file jeb0028-0105-sd3.docx]

Table S3

| Difference chromosome number between eusocial and solitary species | | | | | | | | |
| --- | --- | --- | --- | --- | --- | --- | --- | --- |
| Fixed effects | | | | | Random effects | | | |
| Coefficient | Post.mean | LCI | UCI | pMCMC | Coefficient | Post.mean | LCI | UCI |
| *Taxonomic mixed model (log transformed)* | | | | | | | | |
| Intercept (Eusocial.FALSE) | 2.37 | 2.14 | 2.57 | <0.001 | Superfamily | 0.076 | 0.004 | 0.18 |
| Eusocial.TRUE | 0.091 | -0.092 | 0.258 | 0.308 | Family | 0.037 | 0.0002 | 0.095 |
|  |  |  |  |  | Subfamily | 0.041 | 0.014 | 0.071 |
|  |  |  |  |  | Genus | 0.05 | 0.042 | 0.068 |
|  |  |  |  |  | Units | 0.068 | 0.063 | 0.073 |
| *Phylogenetic mixed model (log transformed)* | | | | | | | | |
| Intercept (Eusocial.FALSE) | 2.51767 | 1.98223 | 3.10401 | <0.001 | Phylogeny | 0.63 | 0.47 | 0.801 |
| Eusocial.TRUE | 0.10 | -0.26 | 0.46 | 0.608 | Units | 0.016 | 0.005 | 0.026 |

Table S4

| Difference chromosome number eusocial continuum | | | | | | | | |
| --- | --- | --- | --- | --- | --- | --- | --- | --- |
| Coefficient | Post.mean | LCI | UCI | pMCMC | Coefficient | Post.mean | LCI | UCI |
| *Taxonomic mixed model (log transformed, continuous, 0=solitary, 1=* Cooperative breeder, 2= Facultative eusocial, 3= Obligate eusocial*)* | | | | | | | | |
| Intercept | 2.38 | 2.17 | 2.59 | <0.001 | Superfamily | 0.082 | 0.007 | 0.20 |
| Eusociality score | -0.0067 | -0.067 | 0.052 | 0.804 | Family | 0.045 | 0.0002 | 0.11 |
|  |  |  |  |  | Subfamily | 0.040 | 0.014 | 0.074 |
|  |  |  |  |  | Genus | 0.053 | 0.040 | 0.068 |
|  |  |  |  |  | Units | 0.068 | 0.063 | 0.073 |
| *Taxonomic mixed model (log transformed, categorical)* | | | | | | | | |
| Intercept (Solitary) | 2.387 | 2.20 | 2.59 | <0.001 | Superfamily | 0.081 | 0.001 | 0.19 |
| Cooperative breeder | 0.132 | 0.17 | 0.426 | 0.354 | Family | 0.031 | 0.0003 | 0.094 |
| Facultative eusocial | -0.156 | -0.50 | 0.124 | 0.324 | Subfamily | 0.047 | 0.016 | 0.083 |
| Obligate eusocial | 0.031 | -0.159 | 0.228 | 0.772 | Genus | 0.053 | 0.040 | 0.067 |
|  |  |  |  |  | Units | 0.068 | 0.062 | 0.073 |

Table S5

| Correlation between chromosome number and colony size | | | | | | | | |
| --- | --- | --- | --- | --- | --- | --- | --- | --- |
| Coefficient | Post.mean | LCI | UCI | pMCMC | Coefficient | Post.mean | LCI | UCI |
| *Phylogenetic mixed model (log transformed), linear effect colony size* | | | | | | | | |
| Intercept | 2.70 | 2.12 | 3.27 | <0.001 | Phylogeny | 0.49 | 0.31 | 0.67 |
| Log(colony.size) | 0.013 | -0.015 | 0.036 | 0.324 | Units | 0.018 | 0.003 | 0.037 |
| *Phylogenetic mixed model (log transformed), polynomial effect colony size* | | | | | | | | |
| Intercept | 2.19 | 1.46 | 2.91 | <0.001 | Phylogeny | 0.51 | 0.33 | 0.69 |
| Log(colony.size) | 0.16 | 0.048 | 0.28 | 0.006 | Units | 0.01 | 0.0004 | 0.026 |
| Log(colony.size)^2^ | -0.008 | -0.0149 | -0.002 | 0.012 |  |  |  |  |
| *Phylogenetic mixed model (log transformed), polynomial effect colony size. Restricted to obligately eusocial species* | | | | | | | | |
| Intercept | 2.32 | 1.16 | 3.42 | <0.001 | Phylogeny | 0.51 | 0.33 | 0.69 |
| Log(colony.size) | 0.14 | 0.017 | 0.27 | 0.026 | Units | 0.01 | 0.0004 | 0.026 |
| Log(colony.size)^2^ | -0.008 | -0.015 | -0.001 | 0.040 |  |  |  |  |

Table S6

| Chromosome number, colony size and colony relatedness | | | | | | | | |
| --- | --- | --- | --- | --- | --- | --- | --- | --- |
| Coefficient | Post.mean | LCI | UCI | pMCMC | Coefficient | Post.mean | LCI | UCI |
| *Phylogenetic mixed model (log transformed), difference between high and low relatedness colonies* | | | | | | | | |
| Intercept (high relatedness) | 2.85 | 2.20 | 3.36 | <0.001 | Phylogeny | 0.439 | 0.255 | 0.60 |
| low relatedness | -0.076 | -0.216 | 0.09 | 0.37 | Units | 0.013 | 0.0006 | 0.027 |
| *Phylogenetic mixed model (log transformed), polynomial effect colony size* | | | | | | | | |
| Intercept (high relatedness) | 1.64 | 0.62 | 2.66 | <0.001 | Phylogeny | 0.46 | 0.30 | 0.62 |
| Log(colony.size) : high relatedness | 0.35 | 0.090 | 0.62 | 0.01 | Units | 0.005 | 0.0002 | 0.013 |
| Log(colony.size)^2^ : high relatedness | -0.02 | -0.04 | -0.003 | 0.024 |  |  |  |  |
| low relatedness | 0.52 | -0.61 | 1.72 | 0.386 |  |  |  |  |
| Log(colony.size) : low relatedness | -0.19 | -0.49 | 0.131 | 0.238 |  |  |  |  |
| Log(colony.size)^2^ : low relatedness | 0.013 | -0.007 | 0.033 | 0.23 |  |  |  |  |

Table S7

| Chromosome number and worker castes | | | | | | | | |
| --- | --- | --- | --- | --- | --- | --- | --- | --- |
| Coefficient | Post.mean | LCI | UCI | pMCMC | Coefficient | Post.mean | LCI | UCI |
| *Taxonomic mixed model (log transformed, categorical)* | | | | | | | | |
| Intercept (No worker caste) | 3.66 | 3.22 | 4.20 | <0.001 | Subfamily | 0.055 | 0.0003 | 0.187 |
| 1 worker caste | -0.92 | -1.37 | -0.458 | 0.002 | Tribe | 0.077 | 0.0003 | 0.173 |
| 2 worker castes | -0.98 | -1.52 | -0. 436 | 0.010 | Genus | 0.027 | 0.0003 | 0.08 |
| 3 worker castes | -0.93 | -1.50 | -0.17 | 0.008 | Units | 0.116 | 0.091 | 0.14 |
|  |  |  |  |  |  |  |  |  |

Table S8

| Difference chromosome number between eusocial and social parasitic ants. | | | | | | | | |
| --- | --- | --- | --- | --- | --- | --- | --- | --- |
| Fixed effects | | | | | Random effects | | | |
| Coefficient | Post.mean | LCI | UCI | pMCMC | Coefficient | Post.mean | LCI | UCI |
| *Taxonomic mixed model (log transformed)* | | | | | | | | |
| Intercept (Eusocial) | 2.75965 | 2.63210 | 2.90410 | <0.001 | Subfamily | 0.031 | 0.0002 | 0.088 |
| Social parasite | 0.21868 | 0.04049 | 0.42403 | 0.016 | Tribe | 0.022 | 0.0004 | 0.059 |
|  |  |  |  |  | Genus | 0.076 | 0.045 | 0.11 |
|  |  |  |  |  | Units | 0.10 | 0.090 | 0.11 |

Table S9

| Correlation between chromosome number and geographic range | | | | | | | | |
| --- | --- | --- | --- | --- | --- | --- | --- | --- |
| Fixed effects | | | | | Random effects | | | |
| Coefficient | Post.mean | LCI | UCI | pMCMC | Coefficient | Post.mean | LCI | UCI |
| *Taxonomic mixed model (log transformed)* | | | | | | | | |
| Intercept | 2.74 | 2.57 | 2.94 | <0.001 | Subfamily | 0.049 | 0.0003 | 0.121 |
| Geographic range | 0.013 | -0.019 | 0.043 | 0.386 | Genus | 0.078 | 0.034 | 0.127 |
|  |  |  |  |  | Units | 0.128 | 0.108 | 0.153 |
|  |  |  |  |  |  |  |  |  |
